# Amygdala self-neuromodulation capacity as a window for process-related network recruitment

**DOI:** 10.1098/rstb.2024.0186

**Published:** 2024-10-21

**Authors:** Guy Gurevitch, Nitzan Lubianiker, Taly Markovits, Ayelet Or-Borichev, Haggai Sharon, Naomi B. Fine, Tom Fruchtman-Steinbok, Jacob N. Keynan, Moni Shahar, Alon Friedman, Neomi Singer, Talma Hendler

**Affiliations:** ^1^ Sagol Brain Institute, Tel Aviv Sourasky Medical Center, Tel Aviv-Yafo, Israel; ^2^ Faculty of Medical and Health Sciences, Tel Aviv University, Tel Aviv-Yafo, Israel; ^3^ Psychology Department, Yale University, New Haven, CT, USA; ^4^ Princeton Neuroscience Institute, Princeton University, Princeton, NJ, USA; ^5^ Sagol School of Neuroscience, Tel Aviv University, Tel Aviv-Yafo, Israel; ^6^ Department of Anesthesia and Critical Care Medicine, Institute of Pain Medicine, Tel Aviv Sourasky Medical Center, Tel Aviv-Yafo, Israel; ^7^ School of Psychological Sciences, Tel Aviv University, Tel Aviv-Yafo, Israel; ^8^ The Center for AI and Data Science, Tel Aviv University, Tel Aviv-Yafo, Israel; ^9^ Ben-Gurion University of the Negev, Be'er Sheva, Israel; ^10^ Dalhousie University, Halifax, Nova Scotia, Canada; ^11^ Department of Neurosurgery, Tel Aviv Sourasky Medical Center, Tel Aviv-Yafo, Israel

**Keywords:** amygdala, neurofeedback, functional MRI, EEG, neuroplasticity, neuromodulation

## Abstract

Neurofeedback (NF) has emerged as a promising avenue for demonstrating process-related neuroplasticity, enabling self-regulation of brain function. NF targeting the amygdala has drawn attention to therapeutic potential in psychiatry, by potentially harnessing emotion-regulation processes. However, not all individuals respond equally to NF training, possibly owing to varying self-regulation abilities. This underscores the importance of understanding the mechanisms behind successful neuromodulation (i.e. capacity). This study aimed to investigate the establishment and neural correlates of neuromodulation capacity using data from repeated sessions of amygdala electrical fingerprint (Amyg-EFP)-NF and post-training functional magnetic resonance imaging (fMRI)-NF sessions. Results from 97 participants (healthy controls and post-traumatic stress disorder and fibromyalgia patients) revealed increased Amyg-EFP neuromodulation capacity over training, associated with post-training amygdala-fMRI modulation capacity and improvements in alexithymia. Individual differenaces in this capacity were associated with pre-training amygdala reactivity and initial neuromodulation success. Additionally, amygdala downregulation during fMRI-NF co-modulated with other regions such as the posterior insula and parahippocampal gyrus. This combined modulation better explained EFP-modulation capacity and improvement in alexithymia than the amygdala modulation alone, suggesting the relevance of this broader network to gained capacity. These findings support a network-based approach for NF and highlight the need to consider individual differences in brain function and modulation capacity to optimize NF interventions.

This article is part of the theme issue ‘Neurofeedback: new territories and neurocognitive mechanisms of endogenous neuromodulation’.

## 1. Introduction

**Figure 1.**
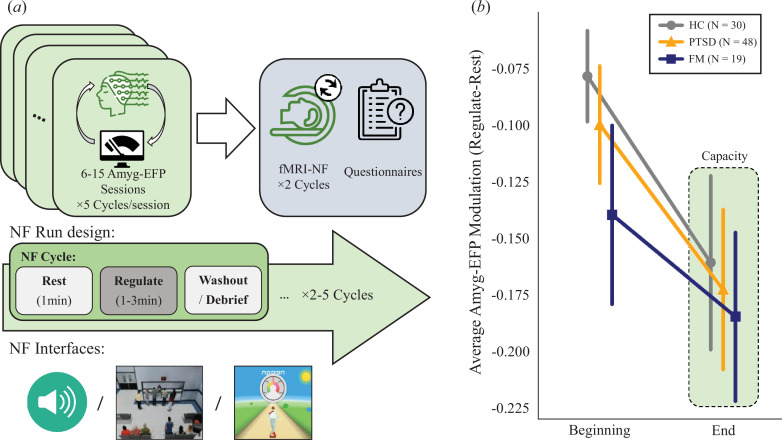
Study design and Amygdala Electrical Fingerprint (Amyg-EFP) capacity overview. (*a*) A schematic of the study design. Participants from three sub-groups of healthy control (HC), post-traumatic stress disorder (PTSD) patients and fibromyalgia (FM) patients went through Amyg-EFP-NF training protocols, followed by post-training assessment, which included fMRI-NF and questionnaires. Each Amyg-EFP session included five cycles combined with interleaved ‘rest’ and ‘regulate’ conditions. Examples of the different NF interfaces used in the study are shown on the bottom row from left to right (auditory, audiovisual waiting room, visual skateboard). (*b*) Average Amyg-EFP modulation averaged at the beginning and final parts of the training. Linear mixed effects model showed a significant effect for training (session). Group differences were not significant. The HC group is shown in grey circles, PTSD in orange triangles and FM in blue squares. The green highlight shows the final Amyg-EFP capacity used in further analyses.

One of the strongest, albeit challenging, ways to demonstrate process-related neuroplasticity is to manipulate an assumed neural mechanism of this process, identify associated neural modifications and test their effect on behavioural and/or psychological indications of this process. Neurofeedback (NF), a non-invasive brain-computer-interface technique, has gained increasing attention in recent years as a potential tool for demonstrating process-related neuroplasticity through self-neuromodulation of one’s brain activity and connectivity. It was further shown that with repeated sessions of training, this reinforcement-based procedure results in an increasing capacity for self-neuromodulation (i.e. defined herby as the difference in the neuromodulation target activation between ‘rest’ and ‘regulate’ conditions; see figure 1) [1,2].

A leading target for neuromodulation in psychiatry has been the amygdala; a major limbic node widely connected to cortical and subcortical regions, which is accordingly involved in multiple mental processes such as fear response [3], anxiety [4,5] and emotion regulation [6,7]. Correspondingly, abnormalities in amygdala activity and its connectivity with other brain areas have been suggested as *trans*-diagnostic markers in psychiatric disorders [8] that are observed, for example, in major depressive disorder [9,10], anxiety disorders [11,12], borderline personality disorder (BPD) [13] and post-traumatic stress disorder (PTSD) [14,15].

The most precise way of modulating the amygdala is via functional magnetic resonance imaging (fMRI)-NF. However, fMRI is costly and largely inaccessible outside of hospital settings, which makes it not scalable. This limitation hinders the optimization of training in terms of the number of sessions and the context in which training takes place. Another established way for targeting neural activity with high temporal precision is electroencephalography (EEG), an accessible and affordable neurophysiological imaging technology. However, the neuroanatomical precision of EEG is questionable, resulting in poor target localization, particularly in deep brain regions such as the amygdala. To advance beyond the state of the art, we have established an analytic way to benefit from both technologies, resulting in fMRI-informed EEG models [16,17]. Such a one-class prediction model is based on the simultaneous acquisition of fMRI and EEG in an independent dataset, and the prediction of well-localized fMRI activity in the amygdala from a time–frequency decomposition of the EEG signal, termed herby amygdala electrical fingerprint (Amyg-EFP); see §2b.

The feasibility and utility of amygdala NF (with fMRI-BOLD or Amyg-EFP) have recently been examined in a meta-analytic review, which showed that individuals can learn to voluntarily change their amygdala activity in real-time and that multiple sessions of amygdala NF have resulted in improved clinical status among patients with depression, anxiety and PTSD [18]. Importantly, it was further reported that amygdala NF led to neural modifications relevant to the emotion-regulation process, such as reduced amygdala reactivity to emotional stimuli [19–24] and enhanced functional connectivity between the amygdala and medial prefrontal cortex (PFC), premotor cortex between and rostral anterior cingulate cortex [25–27].

However, it is well recognized that not all participants are able to regulate their brain activity, including the amygdala, to a similar extent, and that the degree of success in NF training might predict behavioural or clinical outcome of training [28–31]. Indeed, meta-analytic reports of the NF literature reveal significant individual differences in neuromodulation capacity (also referred to as ‘regulation success’), with percentages of poor capacity ranging from 30 to 50% in fMRI-NF studies [18,32] and up to 57% in EEG-NF studies [33].

Understanding the mechanisms of successful self-neuromodulation not only contributes to basic scientific understanding of NF mechanisms but could also inform tailored training strategies that may optimize individual neuromodulation capacity and expand the range of effective clinical applications for diverse patient groups within a disorder category [34].

Previous accounts of the psychological factors that might affect modulation success have highlighted sustained attention and motivation as crucial factors for desired modulation [35], while difficulties in identifying and regulating emotions—as indicated by alexithymia, may hinder it [36]. Methodological factors, such as the type and nature of the interface [37] or the type of instructions [38], were also found to influence modulation success. However, these may depend on the neural target and population studied. While some accounts of neural predictors of neuromodulation success have been made, specifically regarding the target activation levels before training [33], the precise neurobehavioural mechanisms underlying individual self-neuromodulation capacity through NF are yet to be unravelled. Accordingly, it has been debated whether *a priori* neuropsychological conditions (e.g. level of target activation and ability to concentrate) or self-generated acquired skills determine the neuromodulation capacity [32,33,39].

Research has shown that successful neuromodulation was also accompanied by structural [40] and functional [41] neural alterations. fMRI studies have identified specific brain regions and networks that are involved in self-neuromodulation extending beyond the target region, such as the PFC, anterior cingulate cortex and insula [18,42]. These regions have been implicated as the underlying ‘general neural processes’ in NF across targets. However, it is yet unclear if these regions also determine the range of neuromodulation capacity established over training. We recently showed that successful versus unsuccessful downregulation of amygdala activity during one fMRI-NF session involved a restricted set of regions extending beyond the neural target, which are distinct from the aforementioned general NF processes, including the posterior insula, precuneus and ventromedial PFC [18]. However, these findings were based on a single NF session, which is limited in its ability to uncover the full potential of establishing modulation capacity that may be revealed with repeated training.

Using our scalable neural target (Amyg-EFP), we were able to perform studies with 6–15 repeated NF sessions. PTSD [43–45] and fibromyalgia (FM) [31] patients showed improvement in symptom burden, and healthy controls (HC) under chronic stress showed improved alexithymia scores [25]. However, none of the studies that had post-training fMRI-NF assessment was designed or powered enough to examine the underlying neural mechanisms of successful EFP modulation capacity.

Building on this accumulated data of patients (FM and PTSD) as well as healthy participants undergoing repeated sessions of Amyg-EFP-NF (for downregulation) followed by an outcome session of amygdala fMRI-NF, the current study pursued two aims. The first aim was to characterize the establishment of *neuromodulation capacity* and to assess its process relevance following training for amygdala downregulation, as well as its influence by individual differences in neural reactivity and flexibility. We hypothesized that EFP modulation capacity will enhance over training and that this capacity will be associated with neural and process-related mental outcomes. We further expected that neural activity or modulation success at the beginning of training would contribute to the established neuromodulation capacity at the end of training. The second aim was to reveal the neural correlates of successful neuromodulation (i.e. high capacity) by assessing the involvement of regions beyond the target area of modulation during fMRI-NF. We hypothesized that the high capacity of amygdala down-modulation via fMRI-NF will be accompanied by similar modulation in additional regions as previously shown in healthy participants [18] and that by adding these regions to the target modulation, the variance in EFP capacity and its related process changes will be better explained than by the amygdala modulation alone.

## 2. Material and methods

### (a) Participants

In this project, we re-analysed raw data from several studies that took place in the Sagol Brain Institute at Tel-Aviv Sourasky Medical Center between the years 2015 and 2021. All participants gave written informed consent, and the studies were conducted under the approval of the institutional review board. The HC group included 30 male Israeli Defense Forces combat soldiers undergoing basic military training who were a sub-group from a study by Keynan *et al*. [25]. These participants were a part of the Amyg-EFP-NF group in that study that also underwent post-training fMRI. The PTSD group included 48 patients who were clinically interviewed and met the Clinician-Administered PTSD Scale (CAPS-5) [46] criteria for PTSD. In total, 18 patients from a study by Fruchtman *et al*. [44], 20 patients from a study by Fine *et al*. [43] and ten patients from an unpublished cohort who met the screening criteria but underwent a shorter training protocol (six sessions). The FM group included 19 patients with a confirmed diagnosis of FM according to the American College of Rheumatology 2010 criteria [47] from a study by Or-Borichev *et al*. [in preparation]. Other participants from the aforementioned studies were excluded if they were in the study’s control groups (i.e. No-NF) or if they failed to complete at least 2/3 of the assigned training regimen including the post-training fMRI session. Group demographics and personality scores at baseline are described in electronic supplementary material, table S1.

### (b) Amyg-EFP neurofeedback probe

The Amyg-EFP model was previously developed and validated by our laboratory to enable the prediction of localized activity in the amygdala using EEG only [16,48]. This was done by applying machine learning to EEG data acquired simultaneously with fMRI. The procedure resulted in a *time-delay × frequency × weight* coefficient matrix. EEG data recorded from electrode position Pz at a given time-point are multiplied by the coefficient matrix to produce the predicted amygdala fMRI-BOLD activity. The reliability of the Amyg-EFP signal was validated in a new sample of healthy participants who underwent simultaneous EEG-fMRI [49] as well as a sample of patients with BPD [50].

### (c) Amyg-EFP training

After enrolling and signing informed consent, participants were assigned an Amyg-EFP-NF training regimen according to the specific study protocol. Each NF session lasted 40–60 min including preparation time and began with a 3 min baseline recording at rest. Next, each participant performed five NF cycles, comprising three consecutive conditions—passively attending the interface (‘rest’, 1 min), downregulating Amyg-EFP (‘regulate’, 1–3 min) and debriefing by the experimenter that included a performance summary graph and verbal description of the mental strategies employed during that cycle (figure 1*a*). Participants were not informed about the neural target or its association with specific mental processes. Instead, instructions were to freely use mental strategies, allowing individual adoption of the most effective strategies. The interface used during most sessions was an animated audiovisual scenario developed and validated previously [37]. During the ‘rest’ condition, a virtual hospital waiting room became more agitated, as the number of virtual characters standing in front of a receptionist increased, along with the loudness of their voices. During the ‘regulate’ condition, Amyg-EFP power was calculated every 3 s, and feedback at time t was calculated according to the following formula:


$$FB\left(t\right)=\frac{Regulate\left(t\right)-\mu Rest}{\sigma Rest},$$

where μRest and σRest are the average and s.d. of the Amyg-EFP power during the ‘rest’ condition, respectively. Successful downregulation was reflected in as lower level of agitation in the room. The PTSD sub-group from Fruchtman *et al*. [44] had interleaved sessions with an auditory interface (either a musical excerpt or a script of their traumatic experience) during which successful modulation was reflected as lower volume. Key differences between the group protocols are detailed in table 1.

**Table 1.** Amyg-EFP-NF protocols.

sub-group	previous reference	*n*	no. of sessions	interface type	‘rest’ length	‘regulate’ length	no. of cycles/ session
HC	[25]	30	6	audiovisual	1 min	1 min	5
PTSDa	[44]	18	15	auditory/ audiovisual	1 min	3 min	5
PTSDb	[43]	20	10	audiovisual	1 min	3 min	5
PTSDc	--	10	6	audiovisual	1 min	3 min	5
FM	--	19	10	audiovisual	1 min	2 min	8

### (d) Online electroencephalography acquisition and processing

EEG data were acquired using the V-Amp EEG amplifier (Brain Products, Munich, Germany) and BrainCap electrode cap mounted with sintered Ag/AgCl ring electrodes. The Pz electrode was positioned according to the standard 10/20 system with a reference electrode placed between Fz and Cz. Raw EEG signal was sampled at 250 Hz and broadcasted to RecView software (Brain Products) enabling custom real-time processing through a Dynamic-link library (DLL) compiled from MATLAB 2009b, calculating the Amyg-EFP power every 3 s. The Amyg-EFP model was previously developed and validated in our laboratory to enable the prediction of localized limbic activity from EEG only [16,49].

### (e) Electrical fingerprint capacity calculation

From each session of each participant, we calculated a modulation score using the difference between EFP power during ‘regulate’ and ‘rest’ averaged across training cycles. To ensure stability, Amyg-EFP capacity was defined as the average modulation score of each participant’s final three sessions. To account for potential baseline predictors of capacity, the modulation score from the first cycle of the first session was calculated.

### (f) Self-report assessments

Participants were asked to complete translated Hebrew versions of validated self-report questionnaires before and after the training period. Alexithymia was measured using the Toronto Alexithymia Scale (TAS-20), measuring difficulties in expressing and identifying emotions and consisting of 20 items [51]. State anxiety was measured using the State Trait Anxiety Inventory (STAI) consisting of 20 items [52]. The Beck Depression Inventory (BDI) was used to measure affective symptoms [53]. A one-way ANOVA was performed on each of the psychological measures to examine group differences before training. Change in each measure was calculated as the difference between baseline and post-training scores.

### (g) Post-training functional magnetic resonance imaging-neurofeedback

To examine the transferability of the capacity acquired during training, each participant performed an assessment of amygdala downregulation in a different context. The fMRI-NF paradigm was of similar design as in the Amyg-EFP procedure but employed a different interface and was repeated for two consecutive cycles. During the ‘rest’ condition (1 min), an animated figure skated down a rural road at a fixed speed, with a speedometer marking the current speed at all times. During ‘regulate’ (1 min), participants were asked to lower the speed of the skateboard using the same mental strategies they mastered during Amyg-EFP training, with successful downregulation resulting in a slowing of the environment and marked speed. After each regulated condition, a short summary appeared with the average riding speed (9 s), followed by a washout fixation period (30 s). Of note, the FM group performed a task with the same design during the fMRI-NF, but with an audiovisual interface similar to the one used during training.

A subset of patients from the PTSD and FM groups performed a brief, similar design fMRI-NF session before starting the Amyg-EFP-NF training course. Target activity during the ‘rest’ condition was used as a measure for baseline reactivity levels.

### (h) functional magnetic resonance imaging data acquisition and online feedback calculation

Structural MRI and fMRI scans were performed in a 3.0T Siemens MRI system (MAGNETOM Prisma) using a 20-channel head coil. To acquire high-resolution structural images, a T1-weighted three-dimensional sagittal MPRAGE pulse sequence (repetition time (TR)/echo time (TE) = 1860/2.74 ms, flip angle (FA) = 8°, voxel size = 1 × 1 × 1  mm, field of view (FOV) = 256 × 256  mm) was used. Functional whole-brain scans were performed in an interleaved top-to-bottom order, using a T2*-weighted gradient echo planar imaging pulse sequence (HC group: TR/TE = 3000/35 ms, FA = 90°, voxel size = 1.56 × 1.56 × 3  mm, FOV = 200 × 200  mm, 44 slices; PTSD group: TR/TE = 2500/30 ms, FA = 82°, voxel size = 2.3 × 2.3 × 3  mm, FOV = 220 × 220  mm, 42 slices; FM group: TR/TE = 3000/35 ms, FA = 90°, voxel size = 2.3 × 2.3 × 3  mm, FOV = 220 × 220  mm, 46 slices).

During the fMRI-NF session, activity from the right amygdala was delivered as feedback to the participants. The probing of amygdala BOLD for NF was based on a 6 mm sphere in Talairach space in the right amygdala (coordinates, 20, −5, −14) in correspondence to the amygdala BOLD used as a predictor for the Amyg-EFP model [48]. Momentary beta weights of the predefined ROI (averaged across all voxels of the ROI) were extracted online using Turbo Brainvoyager 3.0 (Brain Innovation). The beta weights were then transferred to MATLAB, and feedback was calculated in the same manner as in the Amyg-EFP procedure.

### (i) functional magnetic resonance imaging data pre-processing and analysis

Imaging data was pre-processed with *fMRIPrep* 21.0.2 [54], which is based on *Nipype 1.6.1* [55]. Anatomical T1-weighted images were corrected for non-uniformity, skull stripped and segmented into cerebrospinal fluid (CSF), white matter (WM) and grey matter tissue. Volume-based spatial normalization to standard space (MNI152NLin2009cAsym) was performed through nonlinear registration with Advanced Normalization Tools (ANTs). For the functional images, head-motion parameters were estimated with respect to a BOLD reference volume generated using a custom methodology of *fMRIPrep*. Next, volumes were slice-time corrected to the middle of the repetition time, re-sampled and corrected for head motion. The BOLD reference was co-registered to the T1w reference with boundary-based registration (six degrees of freedom), and pre-processed images were transformed to standard space. The functional scans were smoothed with a 5 mm full width at half maximum Gaussian kernel following a comprehensive examination and recommendations by [56].

First-level analysis was performed in SPM12 (https://www.fil.ion.ucl.ac.uk/spm/) using four task conditions of interest (‘rest’, ‘regulate’, ‘feedback’ and ‘washout’) and additional regressors of no-interest (six head-motion parameters, averaged BOLD signal from a mask of WM and CSF). Additionally, volumes with a calculated framewise displacement larger than 0.9 mm were marked, and motion outlier regressors were added to the model (i.e. scrubbing). Statistical maps were estimated for the ‘regulate’ > ‘rest’ contrast for further analysis.

For each participant, an estimate of amygdala fMRI modulation was extracted using the Marsbar toolbox [57]. The right amygdala region of interest (ROI) was selected using an anatomical mask based on the Automated Anatomical Labelling Atlas [58]. Based on the extracted modulation, high- and low-capacity participants were defined as those with negative (*n* = 58) and positive (*n* = 39) beta estimates, respectively.

Next, a second-level analysis was performed using a two samples design to test the differences between the high- and low-capacity groups. Peak voxel activations were thresholded at *p*(false discovery rate (FDR) = 0.005) < 0.0007 with clusters larger than 50 voxels.

Finally, a second-level one-sample analysis was performed on the high-capacity group, using the modulation score extracted from the amygdala of each participant as a covariate. A whole-brain activation map was created from the covariate estimate, showing regions co-modulating with the region of interest. Peak voxel activations were thresholded at *p*(family-wise error (FWE) = 0.05) < 3 × 10^−7^ with clusters larger than 50 voxels.

Activation clusters from the covariate map were extracted for each participant using the Marsbar toolbox for SPM and used for a regression analysis explaining Amyg-EFP capacity. First, we applied a baseline model using the activation from the right amygdala cluster. Next, we added all activation clusters to a full model. To assess the contribution of each feature in the capacity model, we used the Shapley additive explanations (SHAP) [59] analytical approach. This method is used to explain the output of machine-learning models by calculating the contribution of each feature to a particular prediction (sample). Aggregated SHAP scores can reveal the generally more important features across predictions.

For a final assessment of the relationship between the change in alexithymia score and the estimated capacity, individual activations from the significant clusters were weighted according to the regression model coefficients, resulting in an estimate of the true capacity.

### (j) Statistical analysis

Linear mixed-effect (LME) models were performed in RStudio (v.2022.07.2 using R v. 4.3.3) using the lme4 package [60]. Model comparison was run between competing LME models in order to select the model that explains the most variability. Significance was calculated using the lmerTest package [61], which applies Satterwaite’s method to estimate degrees of freedom and generate *p* values for mixed models. Other statistical tests were performed using JASP (jasp-stats.org; v.0.9.2). Correlations were calculated using Spearman’s correlation coefficient to account for outliers and non-normally distributed data.

## 3. Results

Ninety-seven participants across the three study groups (i.e., HC, FM and PTSD) completed an Amyg-EFP-NF training course (6–15 sessions; see §2), followed by a post-training assessment session that included measurements of their performance with real-time fMRI-NF targeting amygdala downregulation. Amyg-EFP modulation capacity, which is the focus of this study, was defined as the average signal difference between the ‘regulate’ and ‘rest’ conditions across runs in each training session.

Addressing our first aim of characterizing the establishment of NF capacity, we first tested whether the Amyg-EFP-NF modulation capacity improves over the course of training and across the study groups. To account for possible effects owing to the different study protocols and training length, we used an LME model with EFP modulation as the dependent variable and added fixed effects of session (discrete ordinal variable) and group (PTSD, FM or HC). We included the subject and study (a categorical variable to account for the different acquisitions) as random effects, allowing both random slopes as well as intercepts. The model in R was defined as: Capacity ∼ session+group+(1+group+session|subject)+(1+group+session|study) . We found a significant main effect for session (*β* = −0.02, *t* = −3.96, *p* = 0.007), suggesting a linear improvement in neuromodulation capacity with additional training. Figure 1*b* shows the average modulation during the beginning and last parts of the training (averaged across three sessions) for each group. We did not find an effect for group (*p* > 0.45), suggesting similar behaviour across time between healthy and patient populations. Of note, the variance estimators for the different studies were extremely small (<0.00001), and further analyses were performed without considering this factor. As the capacity increases with training, we considered each participant’s end-of-training score (defined as the average of the last three sessions) as their gained neuromodulation capacity for further analysis.

**Figure 2.**
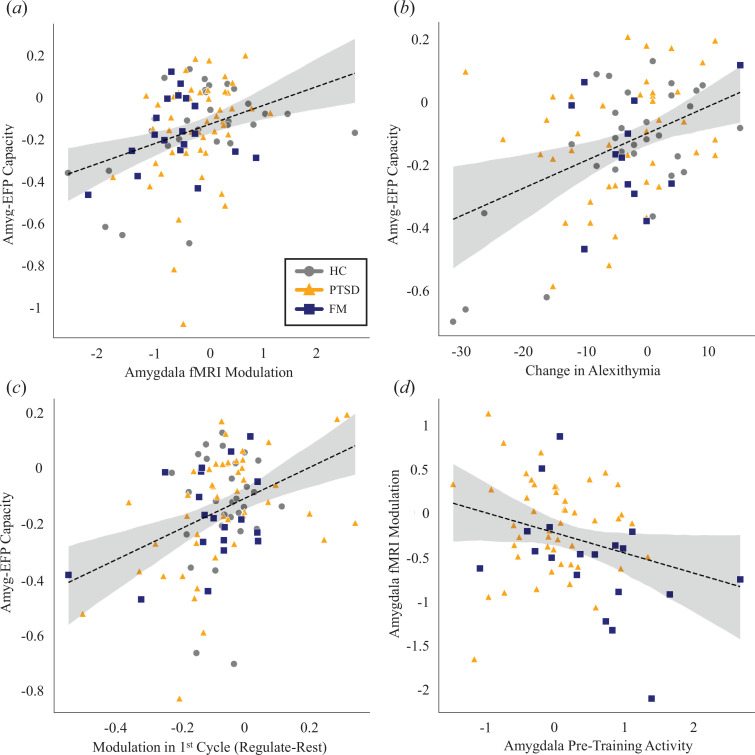
Psychological and neural correlates of capacity. Amyg-EFP capacity correlation is shown with (*a*) Amygdala fMRI modulation during post-training assessment; (*b*) change in alexithymia scale score between pre- and post-training measurements—negative scores imply improvement in identifying and describing emotions; (*c*) Amyg-EFP modulation during the first cycle performed. (*d*) Amygdala fMRI modulation is correlated with pre-training reactivity in a sub-group of the patient who performed pre-training NF run. Reactivity is defined as the amygdala activation during the ‘rest’ condition. Across panels, the HC group is shown in grey circles, PTSD in orange triangles and FM in blue squares.

Next, we sought to evaluate whether the established Amyg-EFP-NF capacity further extends to the neuromodulation capacity of the amygdala, as depicted by the post-training fMRI-NF session. Here, amygdala neuromodulation capacity during fMRI-NF was defined as the contrast in beta estimates between ‘regulate’ and ‘rest’ conditions (regulate > rest), which were extracted from the right amygdala ROI for each participant. No significant differences were found between the study groups (one-way ANOVA: *F*_2_ = 2.25, *p* < 0.1) with a considerable amount of variability noticed within each group. Importantly, linking the two NF modalities, a significant and positive correlation was found between Amyg-EFP-NF gained neuromodulation capacity (as measured during the end-of-training period) and the post-training success in the fMRI-NF (figure 2*a*; *ρ*_97_ = 0.31, *p* = 0.002). This highlights the association between the two measures as indicators of neuromodulation capacity.

We then turned to test the process relevance of the established neuromodulation capacity by examining its association with the training-induced changes in the self-report assessments of anxiety, depression and alexithymia scores. For that, we calculated the difference in each of the measures between questionnaires taken before the Amy-EFP training course and after the fMRI session. As hypothesized, the change in TAS score was associated with Amyg-EFP capacity (figure 2*b*; *ρ*_84_ = 0.31, *p* < 0.015, corrected for multiple comparisons), suggesting that better regulation capacity is related to greater improvement in alexithymia, an emotion-regulation-related mental process that is known to involve amygdala activity and was previously demonstrated to be reduced after NF training for downregulating Amyg-EFP [62]. Negative change scores in this measure suggest an improvement in identifying and describing emotions, alluding to process-related neuroplasticity. Amyg-EFP was not associated with changes in any of the other psychological measures (BDI, STAI *p*’s > 0.2).

To complete the characterization of the establishment of regulation capacity, we examined whether there are neurobehavioural tendencies, already measured at baseline, which may predict the extent of gained capacity. For that, we looked for neural factors measured before training, which could predict the achieved capacity. We examined the very first Amyg-EFP modulation cycle, as a measure for the initial capability to modulate the neural target. We found that stronger modulation in the first run was associated with higher Amyg-EFP capacity following training (figure 2*c*; *ρ*_94_ = 0.358*, p* < 0.001). We then tested the idea that amygdala reactivity, as measured in fMRI, can be also a predictor for regulation capacity. The PTSD and FM study group patients had pre-training fMRI scans with a brief NF run. In these datasets, amygdala activation during the ‘rest’ condition was used as a measure for individual baseline reactivity. As hypothesized, we found that reactivity during rest was associated with amygdala fMRI down-modulation in the post-assessment session (figure 2*d*; *ρ*_66_ = −0.26*, p* = 0.03), indicating that a higher reactivity level may predict better capacity following training.

**Figure 3.**
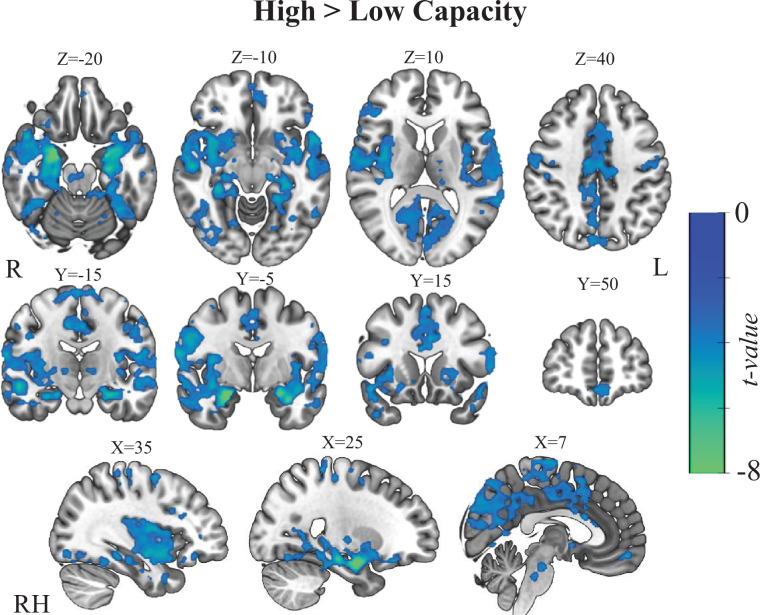
Whole-brain differences between high and low capacity. High- and low-capacity subjects were defined based on average amygdala modulation and directly compared using a two-sample second-level design. Map shows decreased activity in the high- compared to the low-capacity group during the ‘regulate’ > ‘rest’ contrast, for amygdala downregulation. Map is thresholded with *p*(FDR = 0.005) and clusters larger than 50 voxels (see the full table in the electronic supplementary material).

Addressing the second aim of this study, to elucidate the neural underpinning of the gained modulation capacity, we divided the entire sample into high- and low-capacity sub-groups, according to their success in the fMRI-NF session (considering negative beta estimates as successful modulation and vice versa). Figure 3 shows the whole-brain activation maps for the direct comparison between groups (high capacity (*n* = 58) > low capacity (*n* = 39); *p*(FDR = 0.005) < 0.0007, *k* > 50). The map shows extensive regions beyond the expected right amygdala target, including the left amygdala, bilateral hippocampus and parahippocampal gyrus, primary visual areas, bilateral posterior insular cortex, bilateral primary motor cortex, middle cingulate cortex, ventromedial PFC as well as basal ganglia (see the electronic supplementary material, table S2). While these results point to large differences between those succeeding and those who do not, the observed differences may be driven not only by variations in processing related to regulation capacity but also by group differences in the perceptual qualities of the interface, whether given mostly positive or mostly negative feedback, or the subjective experience of success.

**Figure 4.**
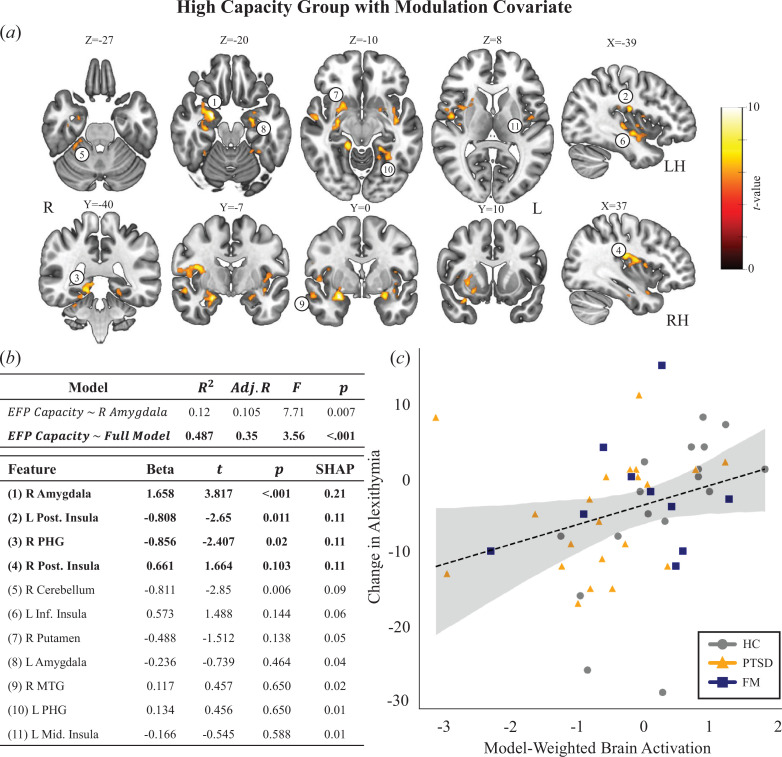
Linear model explaining Amyg-EFP capacity through brain activations. (*a*) Whole-brain activation for the ‘regulate’ > ‘rest’ contrast in the high-capacity group, with a second-level modulation covariate based on amygdala modulation. Map is thresholded with *p*(FWE = 0.05) and clusters larger than 50 voxels (see the full table in the electronic supplementary material). Numbers on the map refer to the full model features. (*b*) Baseline and full model explaining Amyg-EFP variability, with amygdala only or all clusters of activation together. Regions in the full model are ordered according to their SHAP values, indicating their importance to prediction. (*c*) Brain activation weighted according to the full model coefficients is associated with the change in alexithymia score (*ρ* = 0.45; *p* = 0.001).

To account for these possible confounds, we further focused on the high-capacity group and considered the actual capacity of each participant, using a covariate of average amygdala modulation. Figure 4*a* depicts the whole-brain activation map for the ‘regulate’ > ‘rest’ contrast in the high-capacity sub-group with a second-level capacity covariate derived from the average amygdala modulation. This analysis thus focuses on brain regions co-modulating with the right amygdala, which was the target, during neuromodulation. With a stringent threshold corrected for FWE (*p*(FWE = 0.05) < 3 × 10^−7^), a more robust network is introduced, encompassing clusters in the bilateral amygdala, bilateral posterior insula, right parahippocampal gyrus, right cerebellum, right putamen and right temporal regions (see figure 4 legend and the electronic supplementary material, table S3).

This analysis suggests that the activation in additional regions, extending beyond the amygdala target, may support its established modulation capacity. If these regions’ co-modulation is indeed relevant for capacity, this suggests that their involvement would also explain the variance in the Amyg-EFP capacity. To test this question, we used a linear regression model explaining variability in the Amyg-EFP capacity through neural features extracted from this network. As shown earlier, a basic model including only the right amygdala target as a feature for Amyg-EFP capacity is significant and explains 10.5% of the variability (adj. *R* ^2^ = 0.105; *F* = 7.71; *p* = 0.007). Remarkably, when adding all other clusters of activations, the full model significantly increases its explained variability to 35.2% (adj. *R* ^2^ = 0.35; *F* = 3.56; *p* < 0.001; *F*_change_ = 2.92; *p* = 0.005), suggesting that their recruitment is also considerable in explaining the Amyg-EFP modulation capacity (figure 4*b*). To identify the relative importance of each feature compared with others, importance scores were calculated using the SHAP analytic approach [59]. This method, used for explaining machine-learning models by accounting for the effect of each feature on individual predictions, can rank features in the model by order of average importance. We find that the three regions (beyond the right amygdala) with the highest SHAP values are the bilateral posterior insula and right parahippocampal gyrus.

Finally, closing the loop from the neuromodulation back to the process, we asked if the highlighted extended network supporting regulation capacity might further explain the association between the Amyg-EFP modulation capacity and reduction in alexithymia scores. Indeed, the weighted activations from the full linear regression model were highly correlated with the reduction in alexithymia score (figure 4*c*; *ρ* = 0.45; *p* = 0.001), suggesting that the recruitment of this network may support process engagement.

## 4. Discussion

In the current study, we aimed to thoroughly investigate the neurobehavioural underpinnings of ‘neuromodulation capacity’—the degree of successful volitional self-regulation of a specific neural signal through NF training. This neuromodulation capacity, which varies widely among individuals, differs from additional aspects of NF behaviour as learning slopes or pre–post changes and represents an aspect related to the overall aptitude for neural self-regulation. Using a unique dataset from healthy and patient samples, our study combined a relatively extensive Amyg-EFP-NF repeated training period of 6–15 sessions with post-training amygdala fMRI-NF sessions. This combination allowed us to both characterize the Amyg-EFP modulation capacity in terms of its association with the emotional mental process and neuroanatomical underpinnings (first aim) and unveil the broader neural networks as depicted by fMRI, exceeding the target of modulation (i.e. the amygdala), that are involved in successful Amyg-EFP modulation and process modification (second aim).

### (a) Validation of amygdala-electrical fingerprint as a surrogate for limbic neuromodulation capacity

We first demonstrated, as hypothesized, that Amyg-EFP neuromodulation capacity could be improved through training (figure 1). Importantly, we also found that such capacity is strongly associated with its neuroanatomical target modulation, as depicted by post-training amygdala fMRI-NF; a gold-standard procedure for self-neuromodulation of deeply located limbic regions (figure 2; [34,63]). Alluding to an associated psychological process, we further showed that the established capacity to downregulate the Amyg-EFP is associated with greater improvement in alexithymia over training. This highlights the process relevance of Amyg-EFP-NF, whereby its acquired regulation capacity, rather than the one observed at the start, is associated with an improved ability to identify and describe emotions—a process strongly attributed to amygdala function and emotion regulation [64–66]. Demonstrating a reduction in alexithymia following Amyg-EFP-NF complements our previous findings among military first responders [25]. It is particularly interesting in light of the alleviated alexithymia scores observed during chronic stress among combat soldiers [67] pointing to a possible stress inoculation effect of learning to downregulate an amygdala-related neural signal. Considering previous research associating alexithymia with PTSD and combat-related PTSD in particular [68], the current results may further indicate the clinical potential of Amyg-EFP-NF.

These findings collectively support the validity of Amyg-EFP self-neuromodulation as a proxy for limbic modulation across healthy and patient brains. Equally important, these findings highlight the cognitive relevant neuroplastic potential of NF, as reflected by neuromodulation capacity. It shows that self-neuromodulation of limbic activity can be used to induce changes in a relevant mental process of alexithymia, as well as improve the modulation of limbic signal post-training, unravelling effects that persist beyond the period of the Amyg-EFP-NF training.

### (b) Pre-existing neural state and modulation success predict neuromodulation capacity

Next, we showed that although neuromodulation capacity is a malleable measure that can improve through repeated training, neurophysiological markers, such as target reactivity at baseline and modulation ability during the very first trial, provide a good predictor of an individual’s full range of neuromodulation capacity. This finding alludes to two key issues regarding neuromodulation capacity. First, it points to the relevance of baseline target activation as the foundation for established neuroplasticity (i.e. established capacity). This aligns with a recent meta-analysis, which reported a slight positive correlation between pre-training brain activity in the target regions of fMRI-NF during functional localizer runs and learning success [69]. No other ‘general’ brain-based predictors that are consistent across the diverse studies and targets were found in this study. More broadly, our finding of the importance of the target region reactivity further corresponds with previous work showing that synaptic activation is important for neural plasticity [70]. This claim has been also put forth with regard to the efficacy of other neuromodulation techniques in humans such as deep brain stimulation and transcranial magnetic stimulation. It has been shown that the brain state (i.e. context-induced functionality) when the treatment is delivered is an important factor for stimulation outcome [71]. While activity or state dependency in brain stimulation is a well-acknowledged concept, the mechanisms underlying it have not yet been fully unveiled. The precise relationship between ongoing activity and neuronal excitability is probably complex [72,73], and current studies have yielded opposing effects (for a review see Bradley *et al*. [71]). Our exploratory finding that baseline amygdala activation in the fMRI-NF context was associated with greater fMRI modulation capacity is in line with such claims, though needs further investigation. Second, the correlation between neuromodulation ability during the very first attempt and the endpoint neuromodulation capacity points to an *a priori* potential for reaching the maximum neuromodulation capacity. This *a priori* ability or disability has been defined as NF literacy [39], though its trait-like nature has been questioned by others [74,75]. Nevertheless, altogether, the findings pointing to the role of pre-existing neural state and trait suggest that personalized preparation for NF might contribute to the overall achieved neuromodulation capacity.

### (c) The neural basis of neuromodulation capacity extends beyond the amygdala target

The second aim of this study was to investigate the neural underpinning supporting neuromodulation capacity for amygdala downregulation and to investigate which areas beyond the target (i.e. amygdala) may contribute to the established modulation capacity. For that purpose, we analysed the post-training amygdala fMRI-NF data. We first characterized the whole-brain fMRI patterns that distinguish between individuals with high and low capacity in NF neuromodulation of the amygdala. Next, we searched for regions among those with high capacity, where their co-activation with the amygdala during neuromodulation could best explain the established EFP modulation capacity. Previous accounts for the neural correlates of successful neuromodulation found evidence for a role of additional regions in the PFC [76,77] and basal ganglia [78–80], but these were typically limited to within-session contrasts, highlighting the reinforcement learning aspects of the task and to a lesser extent, the acquired skill or the established capacity range. Furthermore, these accounts may be further confounded with processes related to the processing of the feedback or success *per se* [78,81].

The marked categorical differences in brain modulation patterns between high- and low fMRI-capacity groups (figure 4), previously demonstrated in a single fMRI session with a different sample of healthy participants [18], are replicated here in a diverse group of participants (patients and healthy) who underwent extensive Amyg-EFP-NF training prior to the single fMRI-NF session. The results uncovered a broad network of regions, including the posterior insula, ventromedial PFC and hippocampus/parahippocampus. However, the meaning of this broad difference in network recruitment should be interpreted with caution as it could be attributed to general processes related to the NF task, as observed in previous studies [42], or to psychological aspects such as attention allocation and motivation [35]. Furthermore, it could be induced by changes in feedback levels experienced by trainees in the high- and low-capacity groups, meaning observing different scenarios in the feedback interface. In our dataset, this might be particularly worrisome since the interface used in some of the cases was a complex audiovisual scenario, which became quiet and less densely occupied with avatars during successful modulation [37]. To account for these optional explanations, we applied an additional analysis focusing on the high-capacity group in fMRI-NF and used second-level covariate analysis. This analysis identified a more restricted network of regions where neuromodulation, tied to the amygdala, also corresponded with the gained neuromodulation capacity of the Amyg-EFP signal (figure 4). Using an analytical approach to generate feature importance, we further found that the strongest contributing regions to the prediction were the bilateral posterior insula and the right parahippocampal gyrus. These regions are generally found to be functionally connected to the amygdala at rest [82–84] but, intriguingly, also play an important role in shared mental processing related to emotion regulation such as alexithymia [62,85–87]. Of particular interest is the co-modulation of the amygdala with the posterior insula, as both regions are recognized for their roles in processing incoming interoceptive signals related to internal bodily states, such as cardiac and respiratory functions. Notably, an increase in the neural amplification of interoceptive signals, possibly through heightened neural gain of afferent input to these regions, has been linked to an intensified stress response [88]. This finding suggests a potential involvement of interoceptive regulation processes in the success of downregulating the amygdala through NF. However, it is important to acknowledge that such interpretations primarily rely on reverse inference, which infers the presence of specific cognitive processes from observed brain activity patterns. Therefore, we encourage further investigations to build upon these exploratory findings in a hypothesis-driven manner.

### (d) A network-based perspective of neurofeedback

As previously mentioned, investigating the neural mechanism of neuromodulation capacity with respect to a mental process holds promise for uncovering the principles of such cognitive processes. In NF, when brain activity is manipulated in a neuroanatomically precise manner, it offers insights into the functioning of this brain region and its associated network. Unlike traditional lesion studies, NF does not rely on external stimulation hardware, allowing trainees to learn to control their brain activity. This approach aids in understanding the broader dynamics of brain function and facilitates the development of mechanistic theories [89]. Similar to recent findings [90], our finding shows that NF gains significance in the context of connectomics, where manipulating one brain area can impact or recruit a set of distant regions owing to the brain’s complex network structure.

The evolving perspective of the brain as a network, supported by empirical evidence spanning various spatial and temporal scales, underscores the potential for novel insights from unveiling the network beyond the target of modulation (e.g. the amygdala). We believe that establishing a network framework for NF capacity is essential for refining feedback paradigms aimed at identifying neurophysiological drivers of cognition and treating neurological and psychiatric disorders while minimizing adverse effects [91]. This suggestion aligns with a recent network perspective of NF that has been proposed within the framework of network control theory (NCT) [92]. In the context of neuroscience, NCT, which originates from control theory in engineering, is based on the principle that altering the activity of one node would trigger repercussions across the entire network. Additionally, it posits that certain nodes exert more influence over the network owing to their strategic positions within the overall structure. Accordingly, nodes with significant influence, termed control points, can exert varying impacts on the network. NCT suggests that by pinpointing the appropriate set of control points and determining the optimal method to adjust their activity, it becomes feasible to guide the brain from any starting state to a desired target state by applying specific energy patterns to these crucial nodes.

In light of this framework, our findings suggest that repeated sessions of Amyg-EFP downregulation can potentially train individuals to alter an entire network dynamics and to possibly manipulate various aspects of brain network activity, such as flexibility or participation in different functional modules. This opens possibilities for investigating how altering certain nodes with greater influence may exert an impact on the network and associated process, such as emotion regulation, which could greatly enhance our understanding of neurological and psychiatric disorders (see a proof of concept of this idea in Jacob *et al*. [93]). Furthermore, adapting feedback signals based not only on the target but also on network statistics could revolutionize intervention strategies for patients with disrupted functional connectivity patterns.

### (e) Strengths and limitations

While our study benefits from using a large dataset of amygdala downregulation training performed at the same site with multiple training sessions, some limitations should be noted when considering these findings. The use of two distinct patient groups along with HC may hinder our ability to observe symptom-related changes or correlations. We have limited the observation to psychological traits measured with questionnaires in all participants but cannot rule out the contribution of other factors to neuromodulation capacity such as mood, motivation and attention allocation as mentioned before [35]. The different training regimens (e.g. number of sessions and type of feedback) may also add variability to our sample, resulting in different capacity levels in the patients who underwent longer training (figure 1*b*). The added variability of the different feedback interface schemes (i.e. noisy audiovisual scenario or quiet running figure) may also add some variability to some of the analyses like the baseline predictions and group difference. Future studies should control or modulate these factors to determine whether they are associated with specific effects. Nevertheless, this variability in the population, training regime and feedback interface also suggests that the highlighted findings may be more generalizable, surpassing a specific context or state. Our results point to a network of regions harnessed among individuals with high capacity as related to successful neuromodulation; however, these are limited to the neural target of the amygdala during downregulation. Future studies or meta-analyses should explore the possibility that this network is also recruited during other NF training targets and while interacting with different interfaces. Finally, the fMRI session used in this study was relatively short and only treated as an outcome for Amyg-EFP training. This has limited our ability to look for task-modulated functional connectivity changes and should be considered when designing future studies.

## 6. Conclusions

This study presents the idea of *neuromodulation capacity* as an NF outcome separated from learning. Our findings suggest that in the case of downregulating the Amyg-EFP, such capacity is associated with improved alexithymia, reflecting better emotion regulation, a critical domain in mental health. The relevance of pre-training target activation and the recruitment of beyond the target network for successful regional modulation points to a possible mechanism of NF-induced neuroplasticity. These findings support the idea that NF outcomes could be further improved by accounting for a broader brain state before and during the training.

## Data Availability

The data used for statistical analyses and figure creation is available at Gurevitch *et al*. [94]. Unprocessed data will be shared upon reasonable request from the authors. Data is also in the electronic supplementary material online [95].
